# Measuring Workplace Travel Behaviour: Validity and Reliability of Survey Questions

**DOI:** 10.1155/2013/423035

**Published:** 2013-07-16

**Authors:** Nicholas A. Petrunoff, Huilan Xu, Chris Rissel, Li Ming Wen, Hidde P. van der Ploeg

**Affiliations:** ^1^Health Promotion Service, Sydney and South Western Sydney Local Health Districts, Level 9 North, King George V Building, Royal Prince Alfred Hospital, Missenden Road, Camperdown, NSW 2050, Australia; ^2^Prevention Research Collaboration, Sydney School of Public Health, Level 2, Medical Foundation Building, University of Sydney, Sydney, NSW 2006, Australia; ^3^Department of Public and Occupational Health, EMGO Institute for Health and Care Research, VU University Medical Centre, 1007 MB Amsterdam, The Netherlands

## Abstract

*Background*. The purpose of this study was to assess the (previously untested) reliability and validity of survey questions commonly used to assess travel mode and travel time. *Methods.* Sixty-five respondents from a staff survey of travel behaviour conducted in a south-western Sydney hospital agreed to complete a travel diary for a week, wear an accelerometer over the same period, and twice complete an online travel survey an average of 21 days apart. The agreement in travel modes between the self-reported online survey and travel diary was examined with the kappa statistic. Spearman's correlation coefficient was used to examine agreement of travel time from home to workplace measured between the self-reported online survey and four-day travel diary. Moderate-to-vigorous physical activity (MVPA) time of active and nonactive travellers was compared by *t*-test. *Results*. There was substantial agreement between travel modes (*K* = 0.62, *P* < 0.0001) and a moderate correlation for travel time (*ρ* = 0.75, *P* < 0.0001) reported in the travel diary and online survey. There was a high level of agreement for travel mode (*K* = 0.82, *P* < 0.0001) and travel time (*ρ* = 0.83, *P* < 0.0001) between the two travel surveys. Accelerometer data indicated that for active travellers, 16% of the journey-to-work time is MVPA, compared with 6% for car drivers. Active travellers were significantly more active across the whole workday. *Conclusions*. The survey question “How did you travel to work this week? If you used more than one transport mode specify the one you used for the longest (distance) portion of your journey” is reliable over 21 days and agrees well with a travel diary.

## 1. Background

Evidence suggests that reducing car use and increasing use of active travel (public transport, walking, and cycling) to travel to work have benefits for public health [1–5]. There is interest from both transport and public health practitioners in promoting active and sustainable travel. Workplace travel plans are a promising way of addressing this, although the evidence that they may improve employee health is equivocal [6].

Workplace travel plans are strategies for managing travel to, from, and during work. Most travel plans are implemented to alter the transport profile of an organisation. Government websites and reports provide guidance on developing workplace travel plans [7]. They outline a process for developing a travel plan which involves an assessment of the transport modes of employees of the organisation at baseline, which can be used to inform the development of the plan and measure the impacts of the plan over time. Since travel time to work is an important determinant of choice of transport mode, it is also measured. 

Many travel plans implemented in various countries [6, 8, 9] have used similar questions to assess transport profiles. Whilst there are published studies assessing the validity and reliability of survey questions assessing travel mode to school [10, 11], a literature search did not identify published studies assessing the validity and reliability of survey questions on travel mode and travel time to work. The purpose of this study was to examine the validity and reliability of a question to assess travel mode over the past week that is commonly used in surveys for developing workplace travel plans [12], as well as a question used to assess travel time.

The specific research questions of this study are the following.

1. What is the level of agreement between online survey-reported travel mode and time compared with the self-report travel diary?
2. How stable are the self-report survey questions on travel mode and travel time on average over a two-to four-week test-retest period?
3. How physically active are people who have been classified as active or inactive commuters by the travel survey during their journey to work and over an entire day?

## 2. Methods

### 2.1. Study Design

This study was part of a workplace travel survey conducted whilst developing a travel plan for Liverpool Hospital in 2011. A subsample of respondents to the workplace survey of travel behaviour were invited to complete a travel diary for four working days, receiving SMS reminders to complete these each morning and evening. Throughout this period participants wore an accelerometer on their right hip, only taking it off during sleep and water-based activities. Participants then repeated the travel survey online when returning their diary and accelerometer one week later. Twenty participants were unable to recomplete the survey since the survey site was down at the time they returned. 

Approval to conduct the research was provided by the Royal Prince Alfred Hospital Ethics committee, and site-specific assessment and governance approval was provided by Research Ethics and Governance at South Western Sydney Local Health District. All participating staff provided written consent following an explanation of the study.

### 2.2. Geographical Context and Travel Mode of Study Participants

Liverpool is a major centre in an outer metropolitan area of south-western Sydney, Australia. The Hospital is a principal referral teaching hospital. At the time of conducting the study, Liverpool Hospital was well serviced by trains, with two railway stations within ten-minute easy-walking distance. Hospital staff also had access to a bus network close to the main entrance of the Hospital and at a large bus interchange. The interchange at Liverpool train station serviced areas throughout south-western Sydney and beyond this area. 

Although the off-road cycling network in the area did have off-road routes in all directions in a 10 km radius of the Hospital, apart from the route to the north along the railway, there were significant gaps in the cycling network in the immediate surrounds of the Hospital. Most of the surrounding area within a 10 km radius of the hospital is flat. There were 800 car spaces for 3700 FTE staff, and there was significant parking overspill in the surrounds. This overspill had the potential to create significant walking distances for some staff choosing to drive to the Hospital who did not have an on-site parking space.

The online all staff survey was conducted in late September of 2011, and the weather was fine on most days in both weeks the survey was conducted. The weather was typical of Spring in Sydney, being fine with moderate temperatures on most days throughout the study period. The survey found that 83% (*n* = 605) of staff drove alone to work, whilst 11% (*n* = 83) used public transport and 4 (*n* = 28) used active transport modes (walking and cycling) to travel to work. The baseline survey results are described in detail elsewhere [13]. 

### 2.3. Study Participants

From 804 staff who had completed an online all-staff (*n* = 3222) survey, 392 volunteered to participate in additional research and provided their contact details after reading an explanation of what the follow-up would involve and being offered an incentive of entering a prize draw. The list of 392 volunteers was printed in order of time and date of completing the survey. Volunteers were called in the order of this list until a total of 65 participants were recruited with an aim of achieving a sample size of at least 50 for this validation study. Since volunteers were called during work hours and a high proportion were clinical staff (*n* = 109, 60%), many could not be contacted by telephone after reaching number 183 on the list. Of 74 staff who answered the telephone, 87% agreed to participate, and the sample was achieved after reaching volunteer 183 of the list of 392.

### 2.4. Measures

Participants completed the question: “How did you travel to work this week? (If you used more than one form of transport, show the method used for the longest (distance) part of the journey).” The 13 response options for each day of the week were walked, cycled, drove a car, car passenger, bus, ferry, train, taxi, truck, motorbike or scooter, worked at home, other, and I did not go to work. See http://www.activetravel.net.au/professionals/tools for survey. The survey also asked staff to indicate the time in minutes for the longest portion of their trip for each of these working days. Participants were also asked how long their daily trip to work was from their front door to their workplace. This was assessed by an online staff survey.

Public transport, walking, and cycling categories were considered “active travellers” since the public transport commute also included an approximate 10-minute walk to major bus and train interchanges in this location. Car categories were considered “nonactive travellers” since for the majority of car drivers their commute was likely to be inactive.

Participants were categorised as “active travellers” overall if they travelled using an active travel mode on half or more of the working days recorded in their travel diary. 

Participants were instructed on how to complete a four-day travel diary which itemised each trip they made, what travel mode they used, and how long it took. 

Participants were also asked to wear an accelerometer for the same four days they kept their travel diary. The Actigraph GT1M accelerometer (Actigraph, LLC, Fort Walton Beach, FL) is an objective measure of the physical activity and sedentary behaviour and was used as the criterion measure.

 The Actigraph is a single axis accelerometer that records activity counts and steps taken, which were stored every 15 seconds. This enabled the accelerometers to capture short and intermittent bursts of activity, which may be expected during stop-start journeys to work by train, bus, walking, or bike.

Moderate-vigorous physical activity time was calculated using the accelerometer data. The travel diary was used to determine the start and finish of the journey to work for which the MVPA attributed to this trip could then be calculated.

After one week these participants returned their travel diary and accelerometer and redid the online survey before leaving.

### 2.5. Analysis

Data were analysed using SPSS V20.0 (Chicago, Illinois). Travel modes were categorised to car, walking/cycling and public transport. Car included travel mode categories of “I drove alone,” “I was a car passenger,” “truck,” “motorbike or scooter,” and “taxi”. Public transport included “bus,” “ferry,” and “train”. Response categories of “I worked at home,” “other,” and “I did not go to work” were excluded. 

To assess the validity of the self-report online survey, the agreement in travel modes between self-reported survey and travel diary was examined with the Kappa statistic. Spearman's correlation coefficient was used to examine agreement of travel time from home to workplace measured between the self-reported online survey and four-day travel diary. 

The test-retest reliability of the survey questions on travel mode and travel time between the two online surveys over the average 21-day period was assessed using the Kappa statistic. The test-retest reliability of travel time from home to workplace between two self-reported online surveys was determined with Spearman's correlation coefficient.

Statistical analysis of validity and reliability excluded weekends for individual days since there were small numbers of weekend workers. Data for weekend workers were included for the overall statistical analysis.

The comparison of the proportion of the time travelling to work that was spent in MVPA (from the accelerometer data) between active and nonactive travellers was assessed using a two-sample proportion test. The comparison of the mean MVPA time for the whole day between nonactive travellers and active travellers was assessed by *t*-test. Public transport users were grouped with walkers and cyclists as active travellers because the number of public transport users was very small. Moreover, this was also considered appropriate since in this geographical context the journey of public transport users included a substantial amount of walking from a rail or bus interchange to their workplace.

## 3. Results

Data for all 65 participants was used to assess MVPA time. Data for 45 participants was used for the validity and reliability study since the survey site was down when some participants returned to redo the survey.

There was a high proportion (80%) of car users among study participants, and two-thirds of participants lived more than 10 km from their workplace.  Table 1 shows the demographic and transport profiles of the 45 validation study survey recompleters and the 65 study participants whose data was used for assessments of MVPA time. The characteristics of the twenty missed participants were compared to other study participants, and there were no significant differences for any of the demographic variables presented in Table 1.

The validity and test-retest reliability of the self-reported travel survey are presented in Table 2. Overall, there were a substantial agreement for travel mode between self-reported survey question and the travel diary (*K* = 0.62, 95% CI 0.35–0.89) and moderate correlation between the survey and diary for travel time (*ρ* = 0.75, *P* < 0.0001). 

When comparing the survey responses overall at two time points, travel mode from home to work had good reliability (*K* = 0.82, 95% CI 0.57–1, *P* < 0.0001), as did travel time from home to work (Spearman's *ρ* = 0.83, *P* < 0.0001). When comparing travel mode day by day, most results for both validity and reliability study showed moderate-to-good agreement, with only one day each for validity and reliability reaching fair agreement.

The mean MVPA time assessed by the accelerometer during commute time (travel from home to work) for all active travellers was 6.1 minutes (SD 3.0), which is 16% of commute time, compared to 2.6 minutes (SD 2.1), which is 6% of commute time for nonactive travellers. This represented a difference of 4 minutes (95% CI 2.32–5.59). The mean MVPA time for the whole day for active travellers was 62.0 minutes (SD 8.7) versus 37.9 (SD 19.3) for inactive travellers, representing a difference of 24.1 minutes between the two means (95% CI 6.5–41.7). On separating public transport users from walked and cycled, this group travelled for the longest period to work and achieved 14% of their daily moderate-vigorous physical activity during their commute.  Table 3 presents mean travel time from survey, travel diary, and MVPA time from accelerometer by three travel modes.

## 4. Discussion

There were moderate-to-substantial correlations between the online survey questions and the travel diary travel mode and travel time measures. These are meaningful levels of agreement for these statistical tests [14, 15].

The difference in the travel diary responses to travel mode across the week compared with the self-report response to the online survey retest may be explained by recall bias. Participants presented on a Thursday commenced their diary on the next working day and then recompleted the online survey the following Thursday. The survey question asks respondents to recall their travel modes for the past week. Therefore, for most participants Thursday and Friday were the furthest days to recall, and these days showed the weakest association with the travel diary response. 

The discrepancy between travel mode in test and retest survey responses may also be explained by recall bias creating inconsistency in responses. Monday shows the weakest association, and in the test survey Monday would have been the farthest day to recall for most respondents since the survey was delivered on a Thursday. For the retest, Thursday or Friday would have been the furthest, and this may have created some inconsistencies that further exacerbated the poor association. In the test survey, this was done to maximise response rate since a significant proportion of staff did not attend work on Fridays. In the retest, participants were asked to fill in the travel diary for the next four working days, and on returning this diary they were asked to redo the survey and recall the week gone by.

Accelerometers are the “gold standard” objective measure for physical activity in population health research [16]. The accelerometer data used to calculate MVPA time showed that active travellers (defined as walkers, cyclists, and public transport commuters in this study) were significantly more active in their morning commute. This activity represented a significantly greater proportion of their total physical activity for the day, and they were more active overall. It is noteworthy that a high proportion of the participants defined as active travellers in this study were public transport users. These results are highly consistent with a recent review of the literature [1], and they do suggest that promoting use of public transport in medium and large workplaces may be an effective population health strategy for increasing physical activity levels. 

Considering the small sample of walkers, cyclists, and public transport users in the study, we compared their results for MVPA time measure by accelerometers to the results for MVPA time for all staff that were measured using survey questions that have been validated previously [17]. For cyclists the average journey time to work in the survey sample was much shorter than MVPA time overall in the all-staff survey; therefore, these results are unlikely to be reflective of active commuting by bicycle generally and may have biased our results for research question 3 towards the null. However, for public transport users, the MVPA times of study participants were similar to those of all staff. This is logical since the major bus interchange and train stations are located at similar distances of about 10-minute easy-walking distance from the Hospital and most public transport users are likely to walk this distance. Further, many workplaces are located at walking distances from train or bus services that could make significant contributions to physical activity for health, and many have parking close to the door or lifts. Therefore, the comparison for MVPA time between public transport commuters and car drivers is likely to be generalisable to many workplaces.

The Hospital had large parking overspill at the time of the study, and there was potential for drivers to walk similar distances to public transport users from their parking spot to work. However, the average MVPA for drivers in the study was approximately two minutes; therefore, their journey did not include significant amounts of physical activity as they must have parked close to their workplace.

Accelerometers measure activity in the vertical plane; therefore, they can underestimate physical activity from cycling. Due to the small number of cyclists, it is unlikely to have changed the results relating to the difference in physical activity levels of active and nonactive commuters.

A limitation of the study was the small number of public transport commuters and participants who walked or cycled to work. In future research, the limited number of active commuters and public transport users could be overcome by oversampling these groups on recruitment into the study and recruiting more participants overall. However, the strength of the correlations in the tests of reliability and validity, and the simplicity of the questions on travel mode and travel time do provide confidence that the questions are stable and measure what they are trying to measure.

## 5. Conclusions

Whilst the evidence for organisational travel plans improving health is equivocal, they have been described as a promising intervention for increasing employee physical activity levels that are worthy of further research. Although guides exist for conducting workplace surveys for developing travel plans, to our knowledge, none of these questions had been assessed for validity or reliability at the time this study was published. The questions on travel mode and time tested in this study were found to be valid and reliable. Therefore, this study makes a significant contribution to the literature for a promising public health intervention. Whilst the sample size is small, the finding that public transport users accrued significant physical activity levels in their morning commute is consistent with a growing body of research on this topic and does indicate that promoting public transport use shows promise for increasing population physical activity levels [1].

## Figures and Tables

**Table 1:** Characteristics of participants in validity study and MVPA time assessment.

	Validation study survey completers *n* (%)	MVPA time assessment completers *n* (%)
Total	45 (100)	65 (100)
Occupational group		
Administration	11 (24)	19 (29)
Medical	3 (7)	4 (6)
Nursing	12 (27)	17 (26)
Allied Health	6 (13)	11 (16)
Commercial	13 (29)	15 (23)
Occupational type		
Clinical	21 (47)	32 (48)
Nonclinical	24 (53)	34 (52)
Age		
18–34	19 (43)	24 (37)
35–54	23 (52)	36 (55)
≥55	2 (5)	5 (8)
Gender		
Female	37 (82)	55 (83)
Male	8 (18)	11 (17)
Distance from home to work*		
<5 km	8 (18)	12 (18)
5–10 km	9 (20)	12 (18)
>10 km	28 (62)	42 (64)
Main travel mode to work		
Car user	33 (73)	52 (80)
Public transport	7 (16)	8 (12)
Walked or cycled	5 (11)	5 (8)
Shift work		
No	36 (80)	55 (83)
Yes	9 (20)	11 (17)

*Based on postcode of residence.

**Table 2:** Spearman's and Kappa correlations for travel mode and travel time comparing online survey retest to travel diary and initial all-staff online survey.

	Travel mode		Travel time	
	*K* (95% CI)	*P*	*ρ*	*P*
Validity				
Overall	0.62 (0.35–0.89)	<0.0001	0.75	<0.0001
Monday	0.77 (0.45–1.0)	<0.0001		
Tuesday	0.79 (0.56–1.0)	<0.0001		
Wednesday	0.89 (0.68–1.0)	<0.0001		
Thursday	0.63 (0.31–0.94)	<0.0001		
Friday	0.38 (−0.05–0.80)	<0.007		

Reliability				
Overall	0.82 (0.57–1)	<0.0001	0.83	<0.0001
Monday	0.37 (−0.15–0.9)	<0.004		
Tuesday	0.81 (0.56–1)	<0.0001		
Wednesday	0.57 (0.24–0.90)	<0.0001		
Thursday	0.69 (0.37–1.0)	<0.0001		
Friday	0.79 (0.39–1.0)	<0.0001		

**Table 3:** Mean survey, travel diary, and accelerometer outcomes by travel mode.

Mode	Survey		Travel diary		Accelerometer	
	*n* (%)	Time (minutes)	*n* (%)	Time (minutes)	% MVPA*	MVPA time** (minutes)
Car	37 (82)	43	33 (73)	46	6	2.6
Public transport	3 (7)	67	7 (16)	60	14	8.3
Walked/cycled	5 (11)	25	5 (11)	16	24	3.8

*% MVPA is the MVPA time for the journey to work from accelerometer data divided by the total time for the journey to work from travel diary.

**MVPA time in minutes for the journey to work calculated for all 65 participants.
